# Supplementary material for: Far-field coherent thermal emission from polaritonic resonance in individual anisotropic nanoribbons
Source: Nat Commun. 2019 Mar 26;10:1377. doi: 10.1038/s41467-019-09378-5 (PMC6435684; doi:10.1038/s41467-019-09378-5)
Supplement: Supplementary file 1 — Supplementary Information [file 41467_2019_9378_MOESM1_ESM.pdf]

Supplementary information

Far-Field Coherent Thermal Emission from Polaritonic Resonance in Individual Anisotropic Nano-Ribbon

Shin et al.

Supplementary Note1: Extinction Coefficient of SiO_2

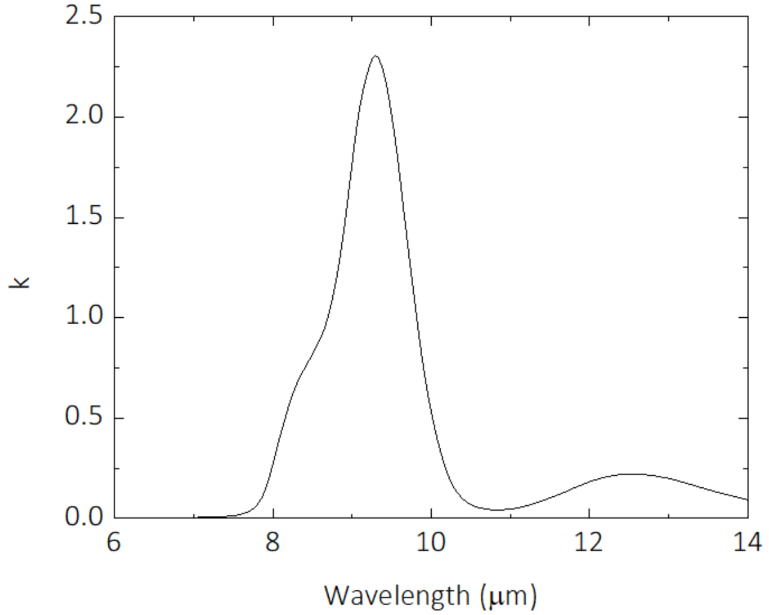

Supplementary Figure 1. A plot of extinction coefficient (k) as a function of wavelength.

Supplementary Note2: Quantifying Radiative Heat Loss

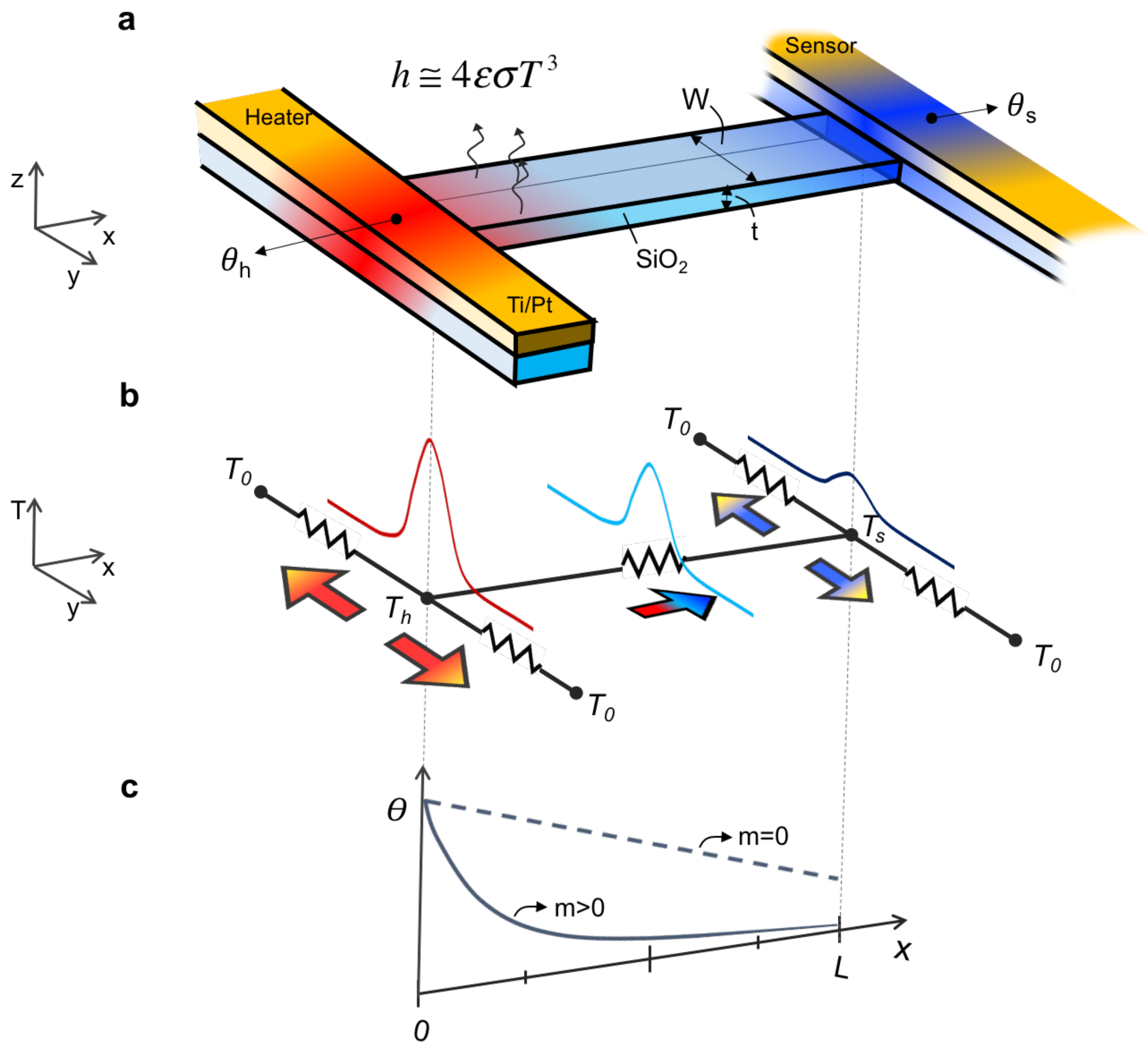

Supplementary Figure 2. (a) Schematic of a SiO_2 ribbon with radiative heat loss ($m > 0$), where θ is a temperature rise. (b) Schematics of temperature profiles along the metal beams (e.g. heater and sensor) and the nanoribbon. (c) Temperature rise along the nanoribbon with and without radiative heat loss are compared (e.g. m>0 and m=0, respectively).

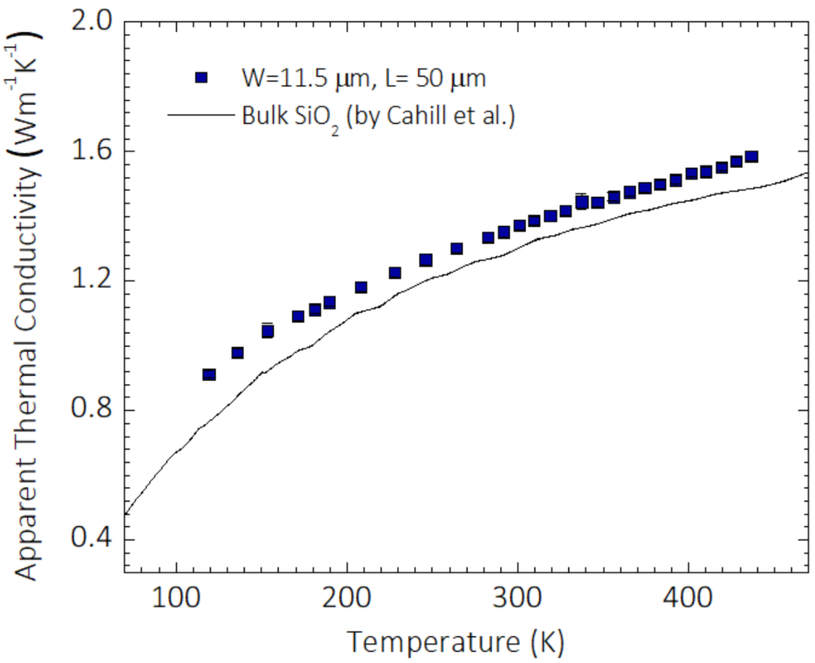

Supplementary Figure 3 Measured thermal conductivity of a nanoribbon (*W*=11.5 μm, *L*= 50 μm and *t*=100 nm) were compared to the bulk SiO_2 thermal conductivity[1].

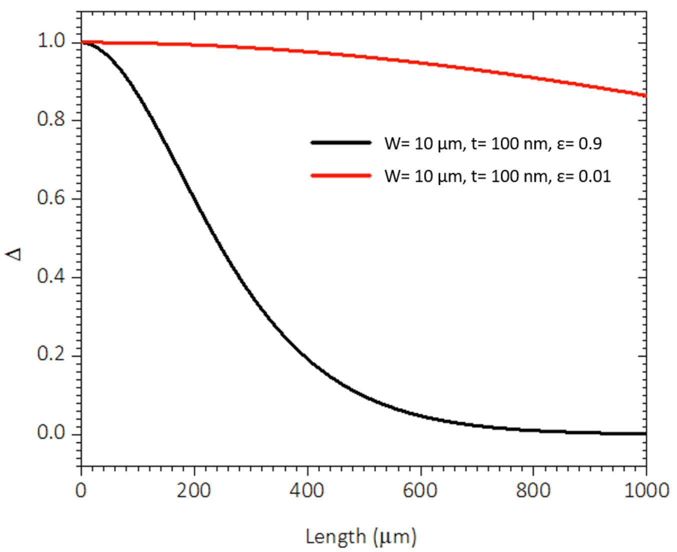

Supplementary Figure 4. Plots of the modeled temperature rising ratio, *Δ* with various lengths at room temperature.

Supplementary Note3: Validation of a Fin Model

We also conducted additional experiments with thick SiO_2 samples where the thickness is larger than the skin-depth, so the thermal radiation would follow the incoherent (broadband) bulk-like emission spectrum. As a result, we would expect the extracted emissivity value to be close to the bulk value of SiO_2 (~0.9 at room temperature). In our new experiments, we extracted the emissivity using the fin model, by following the same procedure as we did for the thin nanoribbons.

We designed the thick SiO_2 beams with 10 μm thickness, i.e., comparable with the skin depth of infrared from 8-10 μm in bulk SiO_2. To determine the suitable beam length to emphasize the radiative heat loss, we estimated the thermal conductance by conduction and radiation with an assumed emissivity of 0.9.

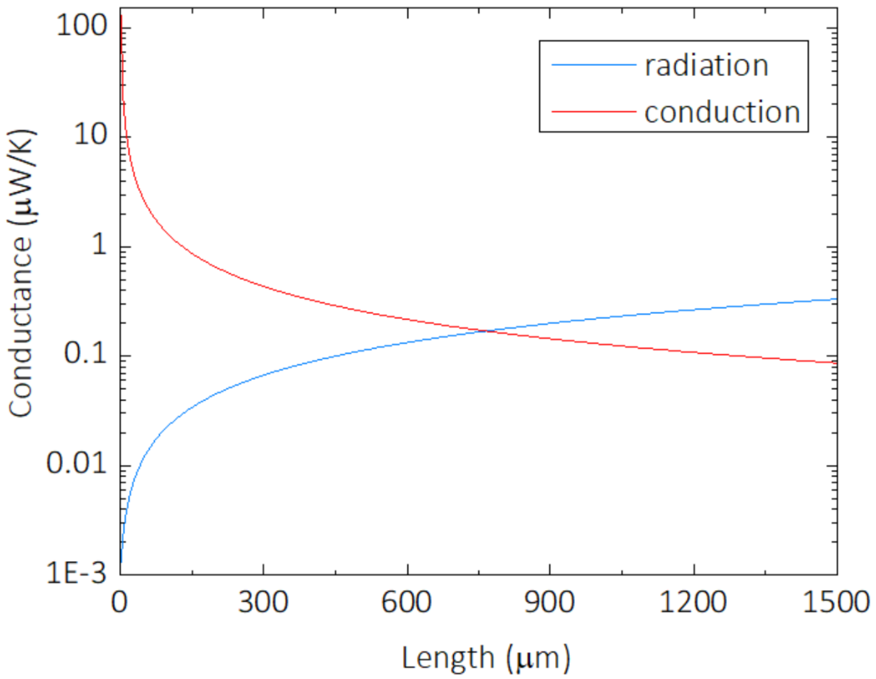

Supplementary Figure 5. Plots of calculated thermal conduction due to phonon conduction and radiation heat loss as a function of the beam length for beam width of 7.5 μm and thickness of 10 μm.

Supplementary Figure 5 shows that radiative conductance would be significant relative to heat conduction when the beam length is around 800 μm or longer. Thus, we fabricated the

suspended SiO_2 beams with various lengths, ranging from 100 to 800 μm as shown in Supplementary Fig. 6.

With various lengths, namely, 100, 400, 600 and 800 μm, we measured thermal conductivity as shown in Supplementary Fig. 7. We analyzed temperature ratios between heating and sensing sides, γ, at each length. At smallest length (100 μm), we obtained thermal conductivity of 1.41 W m^{-1} K^{-1} at room temperature. Again, at this length, the radiation is negligible (see Supplementary Fig. 5) and the measured thermal conductivity agrees well with the expected bulk value of SiO_2. This again validates our heat transfer measurements. By using the thermal conductivity value of the shortest beam or the bulk SiO_2 (~1.4 W m^{-1} K^{-1}), Δ was calculated as shown in Supplementary Fig. 7. Using the same fin model that fits all the sample lengths with a single fitting parameter (i.e., the emissivity), we found the best fit with an emissivity value of 0.77 (±0.07) for the 7.5 μm wide and 10 μm thick beams. This value agrees well with the theoretical expectation. For example, Golyk *et al.* estimated around an emissivity of 0.7 for a cylindrical object with 5 μm radius.[2]

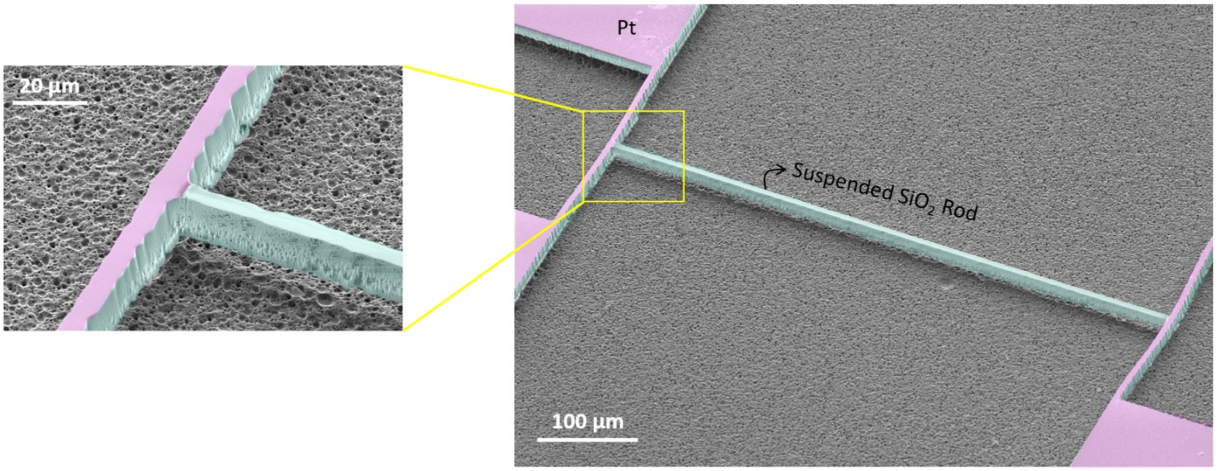

Supplementary Figure 6. A SEM image of a suspended long SiO_2 beam with 10 μm thickness and 7.5 μm width.

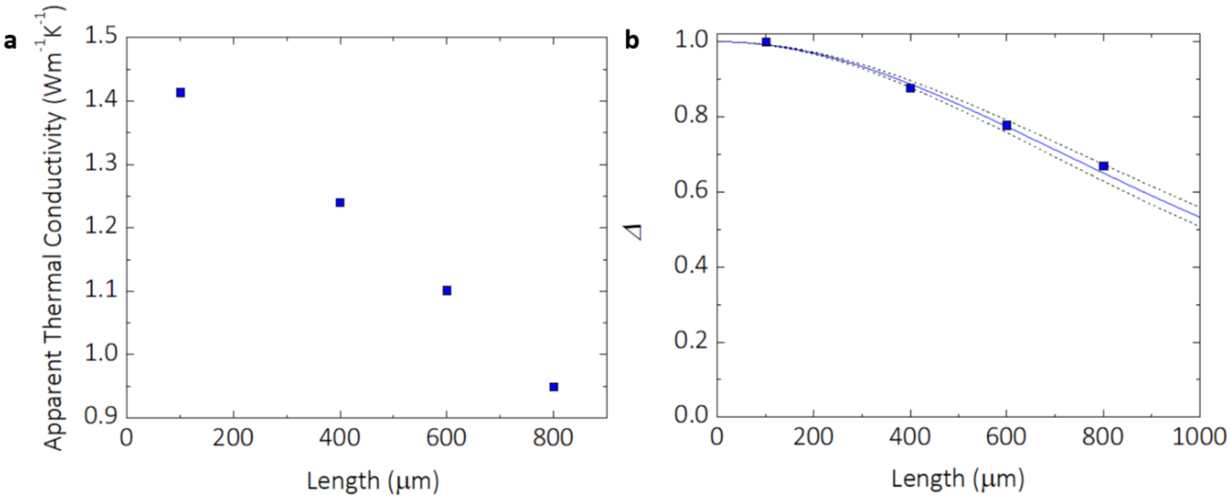

Supplementary Figure 7. Plots of (a) apparent thermal conductivity at room temperature and (b) Δ with various lengths of samples, where the best fit emissivity is 0.77 and the fitting has a standard deviation of 0.07 in absolute emissivity.

Supplementary Note 4: Consideration of Heat Penetration Depth

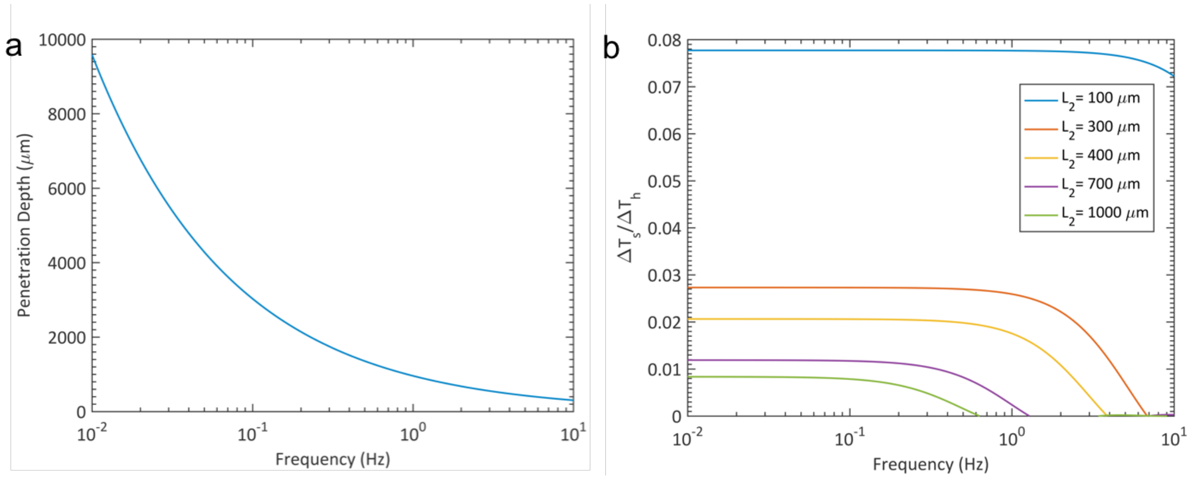

Supplementary Figure 8. Plots of penetration depth (a) and $\Delta T_s/\Delta T_h$ (b) as a function of frequency.

Supplementary Note 5: Measured Temperature rise and Thermal Conductance

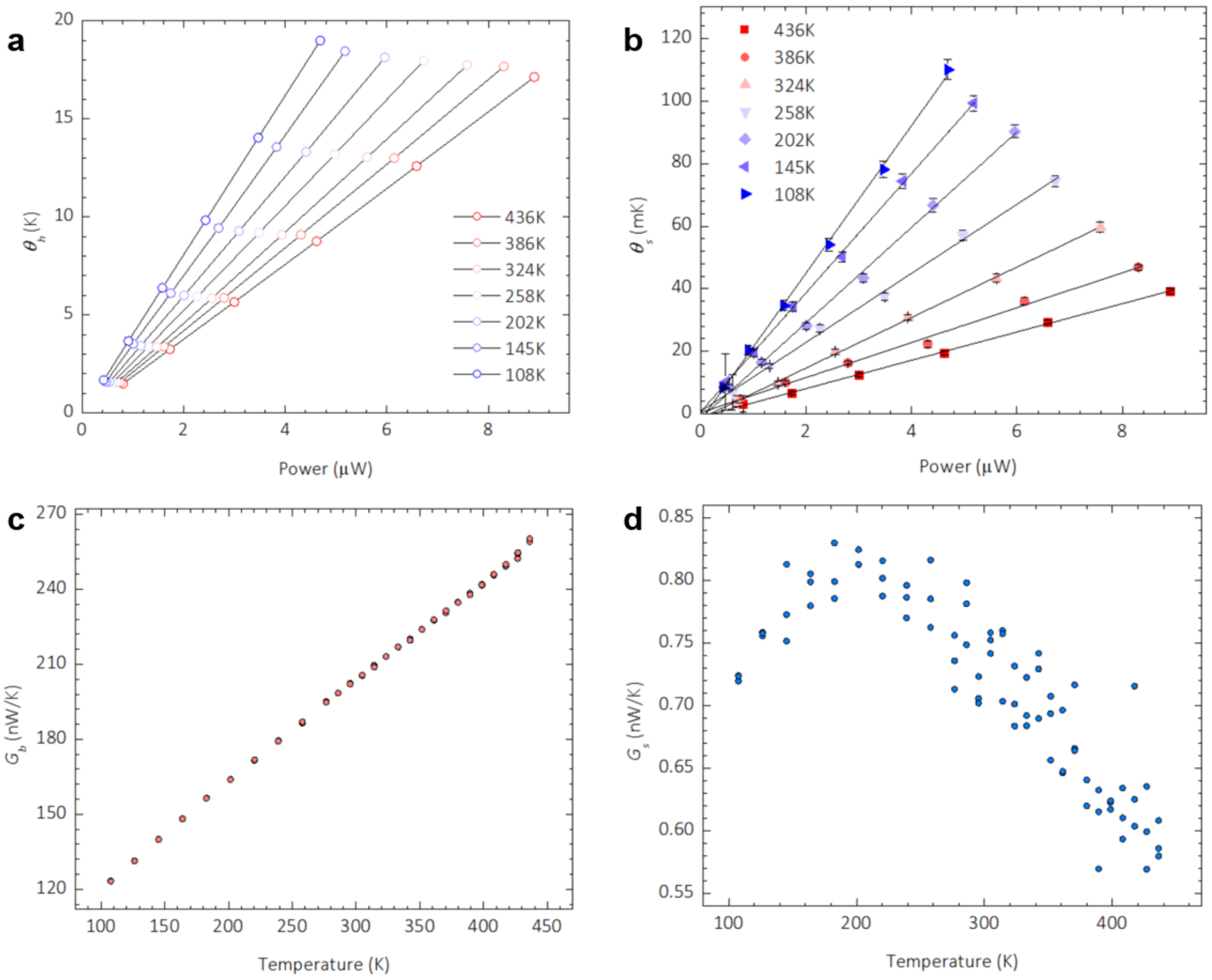

Supplementary Figure 9. Plots of measured temperature rise at the heating (a) and sensing side (b) at different temperatures as a function of heating power, and thermal conductance of the heating (c) and sensing beam (d) as a function of temperature, for the nanoribbon with W=5 μm and L=600 μm.

Supplementary Note 6: Temperature Dependent Heat Transfer Coefficient

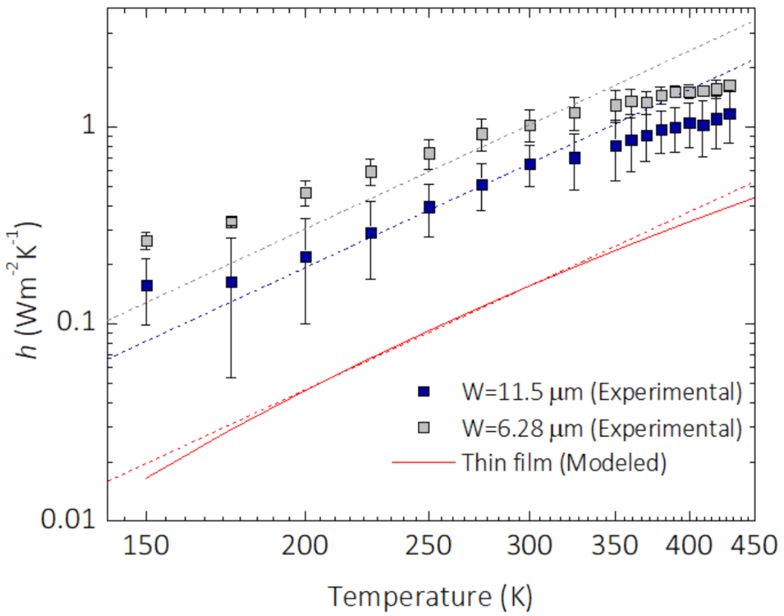

Supplementary Figure 10. Plots of heat transfer coefficient as a function of temperature in log scale. The dashed lines are expected linear h based on the emissivity at 300 K. Error bars corresponds to the uncertainty in the fitting in Fig. 6a.

We analyzed the temperature-dependent emissive behavior. Based on the Planck's distribution, we can expect the heat transfer coefficient, h, is proportional to T^3, and some previous experimental studies have shown the possibility to have different temperature-dependency[3,4] but no specific study has been conducted to reveal the reason behind it. Figure 5a shows indeed different temperature dependency of emissivity between the experimental and the modeling results. The modeling results assumed temperature-independent optical parameter. Therefore, the temperature-dependent behavior is only determined by the peak shift in the Planck's distribution function, and it shows the maximal value at around room temperature corresponding to the thermal wavelength of ~10 μm. We observed nonlinear slope of $\ln{(h)}$ as a function of $\ln{(T)}$, over the broad range of temperature (Supplementary Fig. 10). It implies that the temperature dependency obeys Plank's law by modifying the dielectric constants.

Supplementary Note 7: Emissivity Modeling

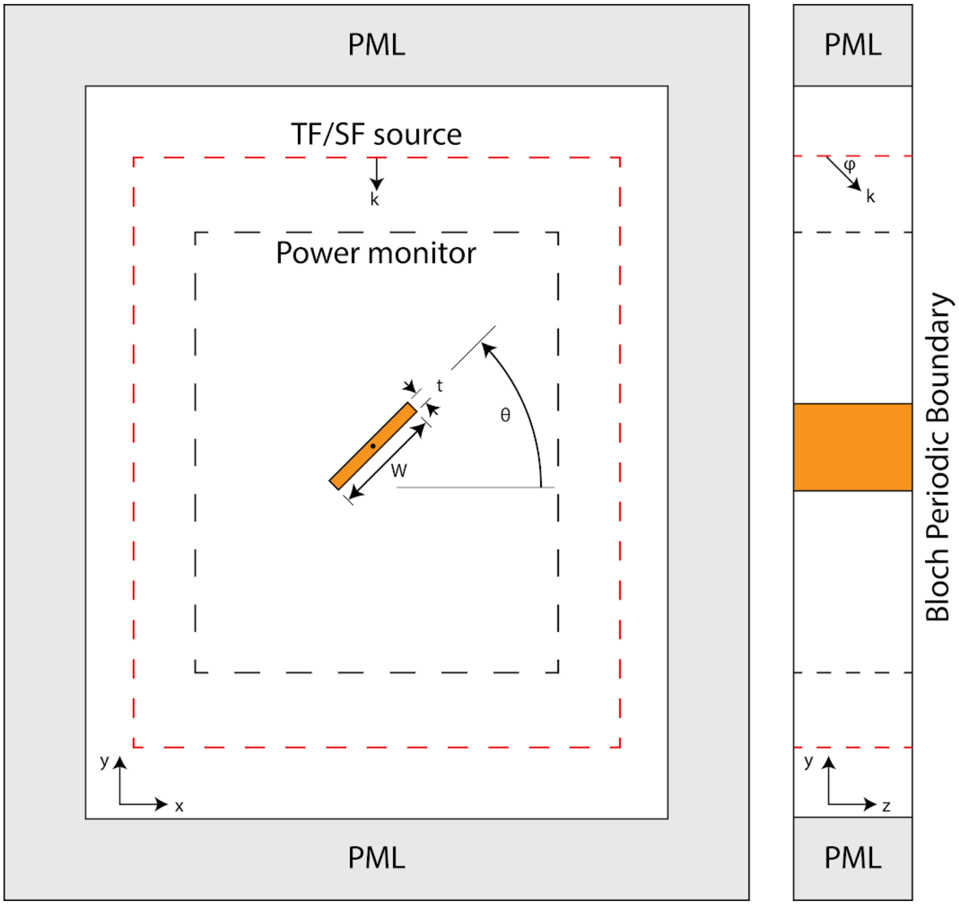

Supplementary Figure 11. Finite-difference time-domain simulation layout.

Supplementary Table 1. Summary of resonant frequencies for SiO_2.[5]

Resonance frequency [$\times 10^{14}$ rad/s]	$\omega_{LO,\,1}$	$\omega_{TO,\,1}$	$\omega_{LO,\,2}$	$\omega_{TO,\,2}$
	0.8	0.9	2.0	2.3

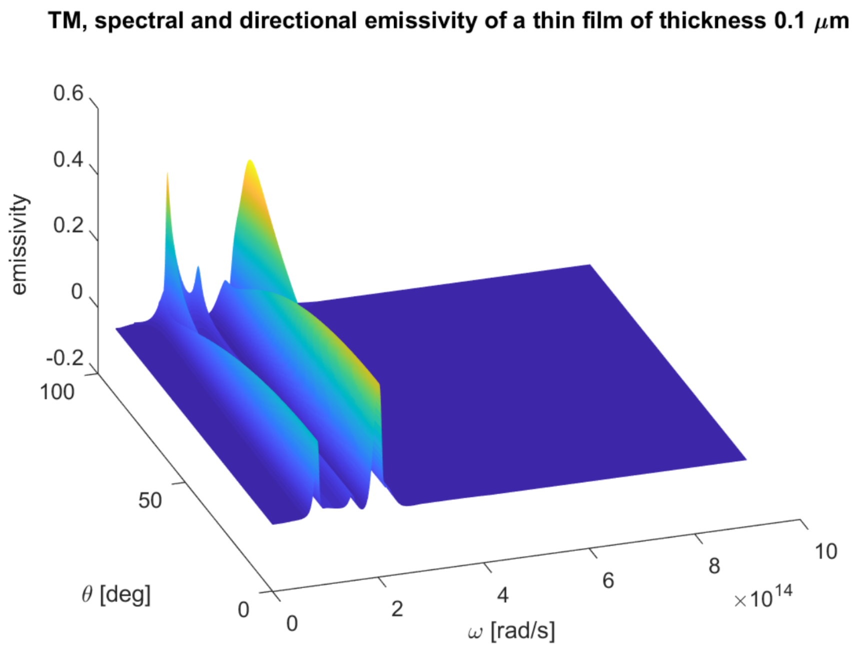

Supplementary Figure 12. Plots of spectral and directional emissivity of TM mode of a 100 nm thin film.

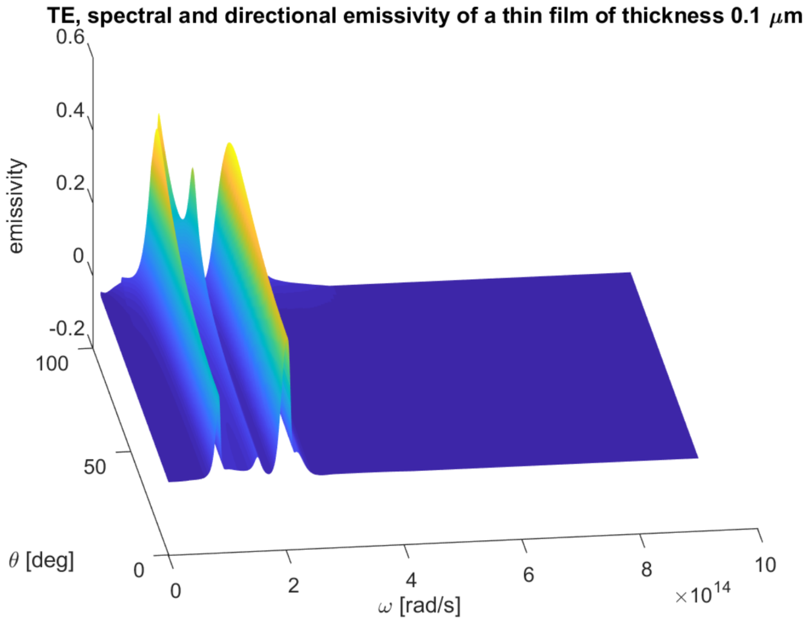

Supplementary Figure 13. Plots of spectral and directional emissivity of TE mode of a 100 nm thin film.

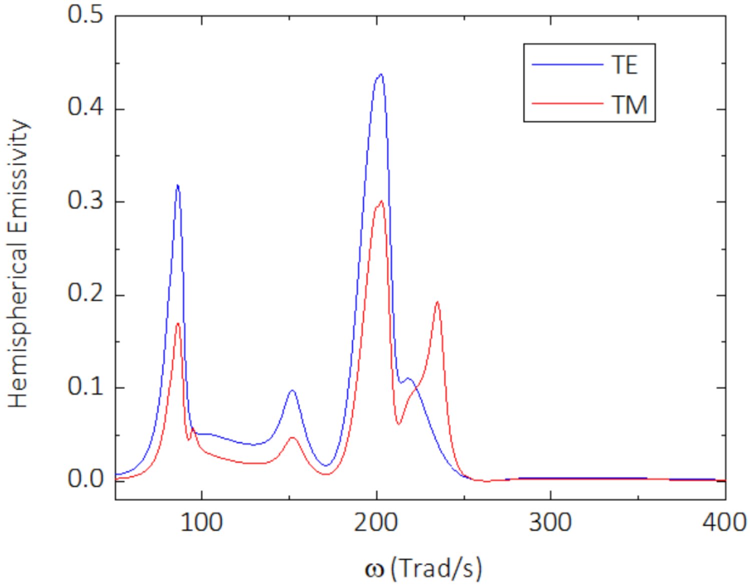

Supplementary Figure 14. Plots of hemispherical emissivity of TE and TM mode of a 100 nm thin film.

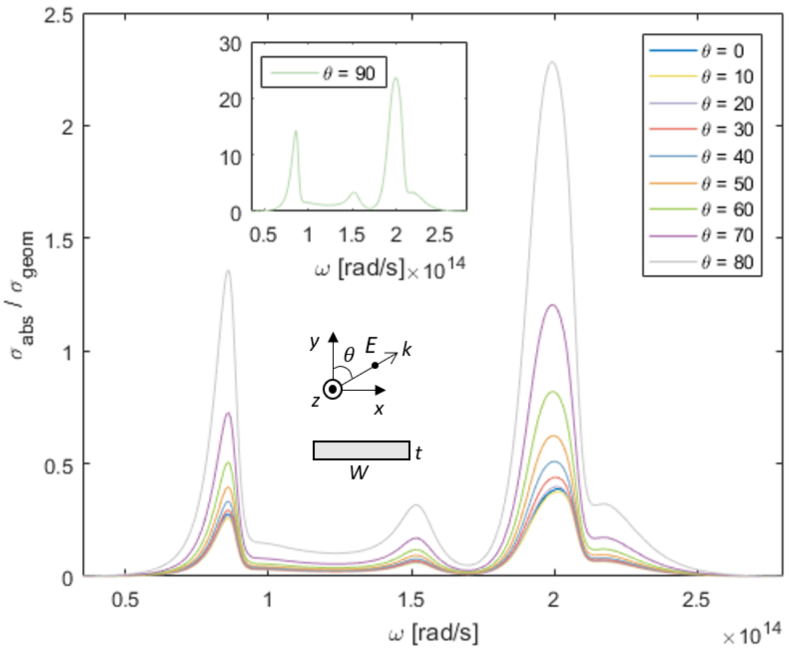

Supplementary Figure 15. Plots of spectral absorption efficiency of nanoribbon (W = 5 μm) with various incident angles, where the polarized electric fields are on the planes including the length.

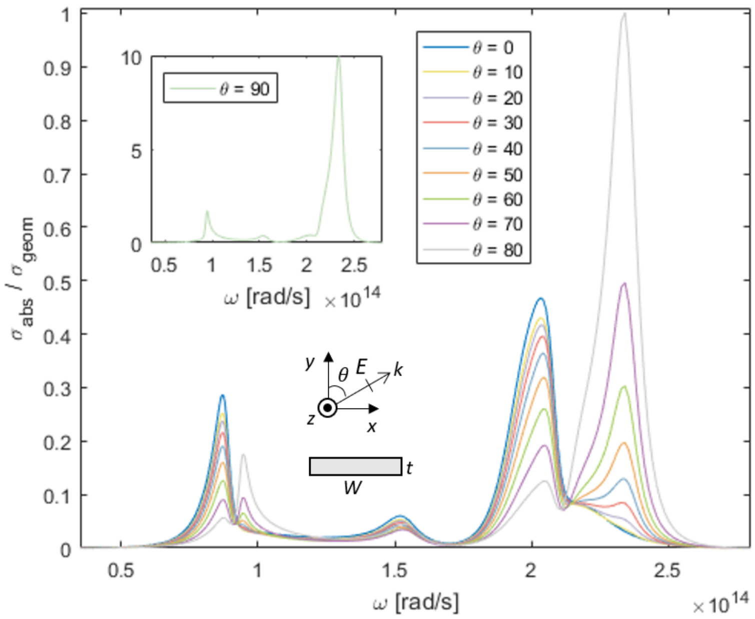

Supplementary Figure 16. Plots of spectral absorption efficiency of nanoribbon (W = 5 μm) with various incident angles, where the polarized electric fields are on the plane normal to the length.

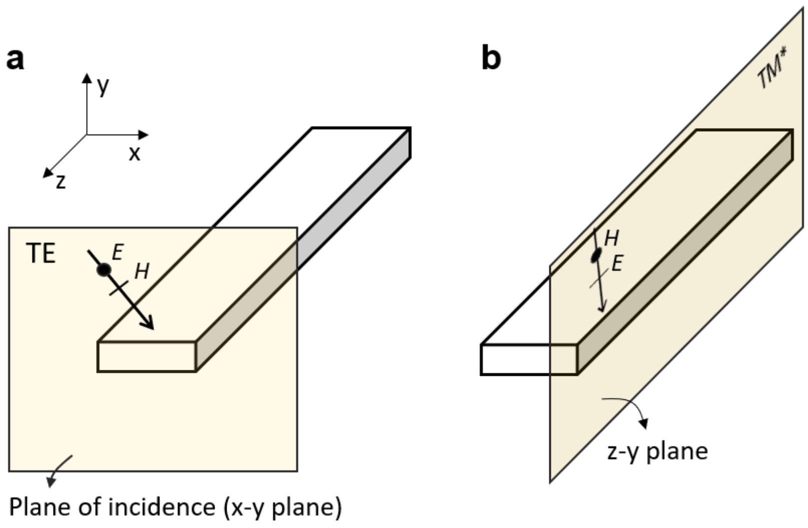

Supplementary Figure 17. Relative polarization modes at different facets.

Our modeling was done with the fixed incident TE or TM polarized wave while rotating the ribbons by different angles. Unlike the infinite film which has only one finite dimension in thickness, the nanoribbon structures have two finite dimensions in both width and thickness. Therefore, although we have an incident wave with the TE or TM polarization for one specific facet, the other perpendicular planes will meet a different polarization, relatively defined by φ and θ.

For instance, here we draw an explicit case where it showed the opposite polarizations with the rotated structures. Supplementary Figure 17a shows incident TE wave on the x-y plane, which is normal to the length. On the plane of incidence, the TE wave does not have an electric field component which is polarized normal to the surface (width or thickness) on x-y plane. On the other hand, as the nanoribbons are 3D structures, we can move our view point to one of perpendicular planes to see how different faces are affected by the TE incident on x-y plane. Supplementary Figure 17b shows the example on the z-y plane. In this normal plane to x-y plane, there is an electric field component polarizing across the surface. Therefore, one incident wave can excite both TE and TM modes in the 3D structures.

Supplementary Note 8: Dispersion Relation

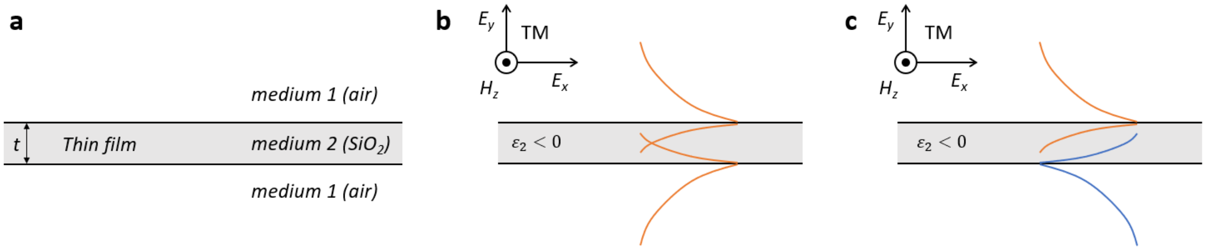

Supplementary Figure 18. Schematics of (a) a thin film structure consisting of a medium 2 (SiO_2) surrounded by medium 1 (air), and (b) symmetric and (c) asymmetric configurations of TM polarized surface waves.

Electromagnetic interactions between two adjunct surfaces can be significant with the poles of the reflective coefficient, *r*, determined by the Fresnel equation,

$$r_{\mathrm{TM}} = \frac{\varepsilon_2 k_{\mathrm{t},1} - \varepsilon_1 k_{\mathrm{t},2}}{\varepsilon_2 k_{\mathrm{t},1} + \varepsilon_1 k_{\mathrm{t},2}} \qquad (1)$$

where ε_i is the complex permittivity, $k_{t,i}$ is the transverse vector ($k_{\mathrm{t,i}}^2 + k_{\mathrm{p}}^2 = \varepsilon_{\mathrm{i}} k_0^2$) in the medium *i*, k_p is the propagating vector along the surface ($k_{\mathrm{p}} = q + i\kappa$), where q and κ are the real and imaginary part of the momentum vector, respectively), and k_0 is the free space vector. To figure out the fundamental modes with two surfaces, we solve

$$1 - r^2 e^{2ik_{\mathrm{t},2}t} = 0. \qquad (2)$$

The above equation yields solutions for the poles of the complex reflectivity. TM polarization can support surface phonon polaritons when the real part of permittivity is negative. The analytical solutions for the above equations can be achieved by considering two different configurations of surface waves at each one of the two interfaces. For the symmetric electric field configuration of TM polarization as shown in Supplementary Fig. 18b,

$$r_{\mathrm{TM}} = e^{ik_{\mathrm{t},2}t} = \frac{\varepsilon_2 k_{\mathrm{t},1} - \varepsilon_1 k_{\mathrm{t},2}}{\varepsilon_2 k_{\mathrm{t},1} + \varepsilon_1 k_{\mathrm{t},2}} \qquad (3)$$

$$\left(\varepsilon_2 k_{\mathrm{t},1} + \varepsilon_1 k_{\mathrm{t},2}\right) e^{ik_{\mathrm{t},2}t} = \varepsilon_2 k_{\mathrm{t},1} - \varepsilon_1 k_{\mathrm{t},2} \qquad (4)$$

$$\varepsilon_2 k_{t,1}\left(1-e^{ik_{t,2}t}\right)=\varepsilon_1 k_{t,2}\left(1+e^{ik_{t,2}t}\right) \qquad (5)$$

$$\frac{\varepsilon_2}{\varepsilon_1}=-\frac{k_{t,2}}{k_{t,1}}\coth\left(\frac{t}{2i}k_{t,2}\right). \qquad (6)$$

In the thin film regime ($k_{t,2}t \ll 2$), the equation can be simplified in the form of:

$$\frac{\varepsilon_2}{\varepsilon_1}=-\frac{2i}{t}\frac{1}{k_{t,1}}. \quad (7)$$

where $k_{t,1}$ is positive. In the case where the highly confined guided modes ($k_{t,i}=\sqrt{\varepsilon_i k_0^2-k_p^2}\approx ik_p$) are supported, the further simplified form follows:

$$k_p=-\frac{2}{t}\frac{\varepsilon_1}{\varepsilon_2}. \quad (8)$$

Note that this algebraically simplified equation only counts the zeroth order mode. With the consideration of the Fabry-Perot resonance, the equation can be shown as[6]:

$$k_p=q(\omega)+i\kappa(\omega)=\frac{i}{t}\left[2\arctan\left(i\frac{\varepsilon_1}{\varepsilon_2}\right)+\pi l\right] \quad (9)$$

where l is an integer for the higher order modes, and the dispersion curves of fundamental modes were shown in Supplementary Fig. 19 with l=0.

Similarly, for the asymmetric electric field configuration for TM polarization as shown in Supplementary Fig. 18c,

$$r_{TM}=-e^{ik_{t,2}t}=-\frac{\varepsilon_2 k_{t,1}-\varepsilon_1 k_{t,2}}{\varepsilon_2 k_{t,1}+\varepsilon_1 k_{t,2}} \quad (10)$$

$$\frac{\varepsilon_2}{\varepsilon_1}=-\frac{k_{t,2}}{k_{t,1}}\tanh\left(\frac{t}{2i}k_{t,2}\right). \qquad (11)$$

In the thin film regime ($k_{t,2}t \ll 2$), the aforementioned equation can be simplified in the forms of:

$$\frac{\varepsilon_2}{\varepsilon_1}=-\frac{t}{2i}\frac{k_{t,2}^2}{k_{t,1}} \quad (12)$$

where $k_{t,1}$ is positive.

By comparing the simplified equations from symmetric and asymmetric dispersions, it is clear that two distinct fundamental modes have opposite thickness-dependent behaviors. While the symmetric mode possesses more bounded modes as the film thickness decreases, the asymmetric

mode becomes closer to the light line with decreasing film thickness, as shown in Supplementary Figs. 19a-b. The thickness-dependent behaviors are clearly shown in the Reststrahlen band ranging from 200 to 220 Trad/s, and the features become deviating from the light line with the dominant confinement effect on the surface. Therefore, we believe it is plausible that surface phonon polaritons are supported. The shrunk wavelength along the surface, unlike the free-space wavelength, efficiently enhances the absorption cross-section. Correspondingly, the higher confinement (high q) yields shorter propagating length (Supplementary Fig. 19c), which implies more efficient absorption.

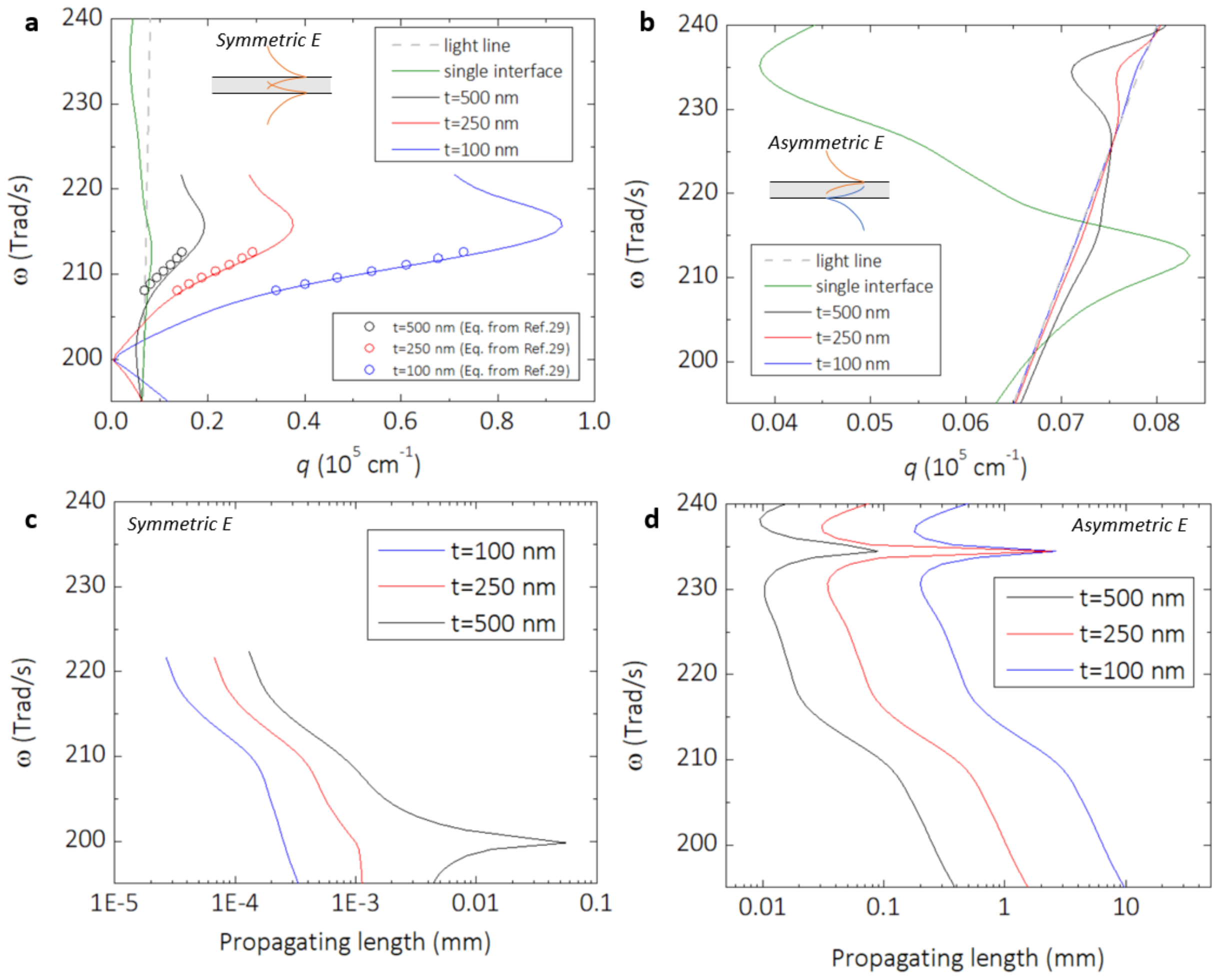

Supplementary Figure 19. Dispersion relations of thin films with various thicknesses, in the cases of symmetric (a) and asymmetric (b) configurations of electric fields. (c,d) Plots of

propagating length, $1/(2\kappa)$, of symmetric (c) and asymmetric (d) modes, respectively. TM polarization was considered as described in Supplementary Fig. 18.

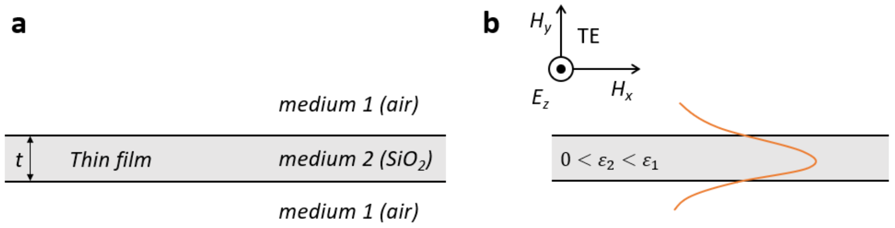

Supplementary Figure 20. Schematics of (a) a thin film structure consisting of a medium 2 surrounded by 1, and (b) TE wave guide mode.

TE polarization does not have an electric field component normal to the surface as shown in Supplementary Fig. 20, thus it cannot support surface waves unless permeability, μ has negative values to form polaritons by magnetic fields in an infinite slab while it still makes reflection at the interface as:

$$r_{\mathrm{TE}} = -\frac{\mu_2 k_{\mathrm{t},1} - \mu_1 k_{\mathrm{t},2}}{\mu_2 k_{\mathrm{t},1} + \mu_1 k_{\mathrm{t},2}}, \text{ where } \mu_1 = \mu_2 = 1. \qquad (13)$$

The only available solution to satisfy positive $k_{\mathrm{t},1}$ is following:

$$r_{\mathrm{TE}} = -e^{ik_{\mathrm{t},2}t} = -\frac{k_{\mathrm{t},1} - k_{\mathrm{t},2}}{k_{\mathrm{t},1} + k_{\mathrm{t},2}} \qquad (14)$$

$$1 = -\frac{k_{\mathrm{t},2}}{k_{\mathrm{t},1}} \tanh\left(\frac{t}{2i} k_{\mathrm{t},2}\right) \qquad (15)$$

$$1 = -\frac{t}{2i}\frac{k_{\mathrm{t},2}^2}{k_{\mathrm{t},1}}, \text{ in the thin film limit } (k_{\mathrm{t},2} t \ll 2). \qquad (16)$$

The analytically calculated dispersions were shown in Supplementary Fig. 16(a). There is a sharp change in the propagating vector, q at around 200 Trad/s, deviating from the light line. More specifically right below ω_{LO} (200 Trad s^{-1}), the real part of $\varepsilon_{\mathrm{SiO_2}}$ becomes smaller than that of air ($\varepsilon_{\mathrm{air}}$=1) down to 199 Trad s^{-1}. In this regime, the repeated internal reflections within the SiO_2 can generate the guided modes in the SiO_2. However, the effective energy range is very narrow to meet $\varepsilon_{\mathrm{SiO_2}} < \varepsilon_{\mathrm{air}}$, furthermore, the thin structure diminishes the guiding effect. The lowest order of the

guide modes requires a half of the wavelength at least in dimensions. In addition to the guiding effect, the SiO_2 also can be considered as a lossy dielectric layer with high refractive index, especially at around 190 Trad/s because of the peaks of both real and imaginary parts of permittivity. Therefore, the effective refractive index of a thin film surrounded by air will be closer to that of air with smaller lossy volume.

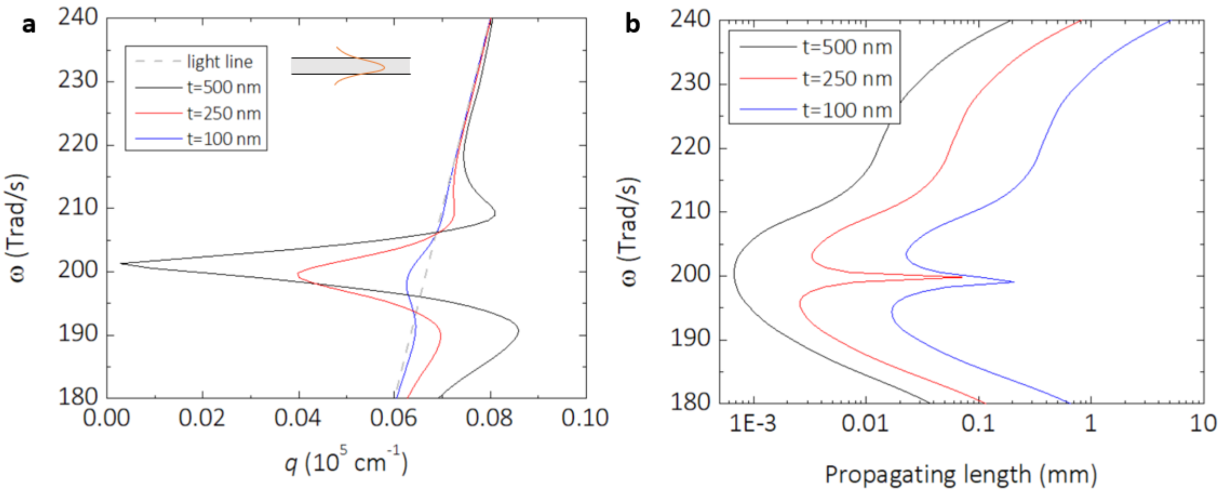

Supplementary Figure 21. (a) Dispersion relations and (b) propagating length of thin films with various thickness, in the case of TE polarized wave-guided modes.

Based on the mode analysis from a 2D infinite slab, it is concluded that the confinement effect with a small thickness (e.g. 100 nm in our experiments) is highly dominant by symmetric modes of TM polarization. The smaller wavelength of surface phonon polaritons, compared to that of free space, enhances absorption efficiency, which is determined by the ratio of absorption cross-section to geometric cross-section. Owing to the shrunk wavelength along the surface (λ_{SPhP}), the absorption efficiency increases with the thinner thickness within the Reststrahlen band. On the other hand, in the regime where the SiO_2 behaves as a lossy dielectric absorber, rather than a metal, it shows higher absorption with the larger volume.

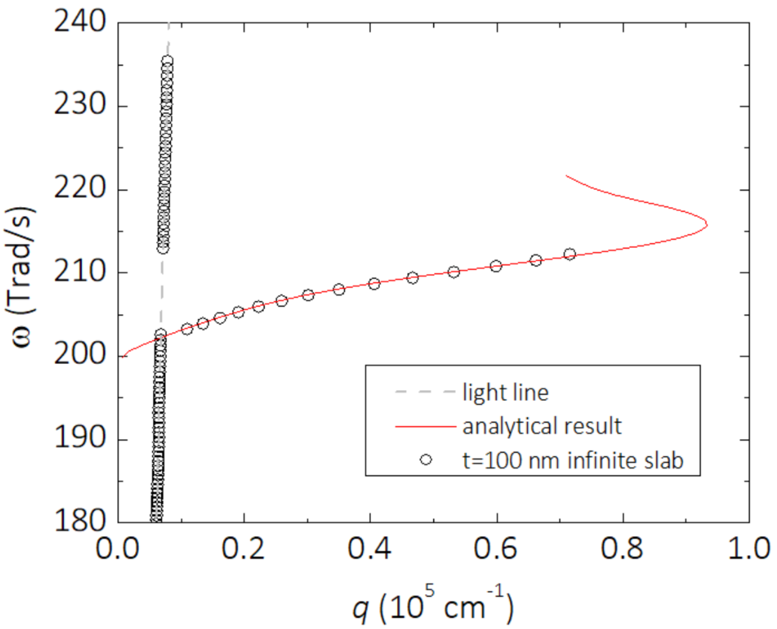

Supplementary Figure 22. Dispersion of an infinite slab by numerical modelling with 100 nm thickness.

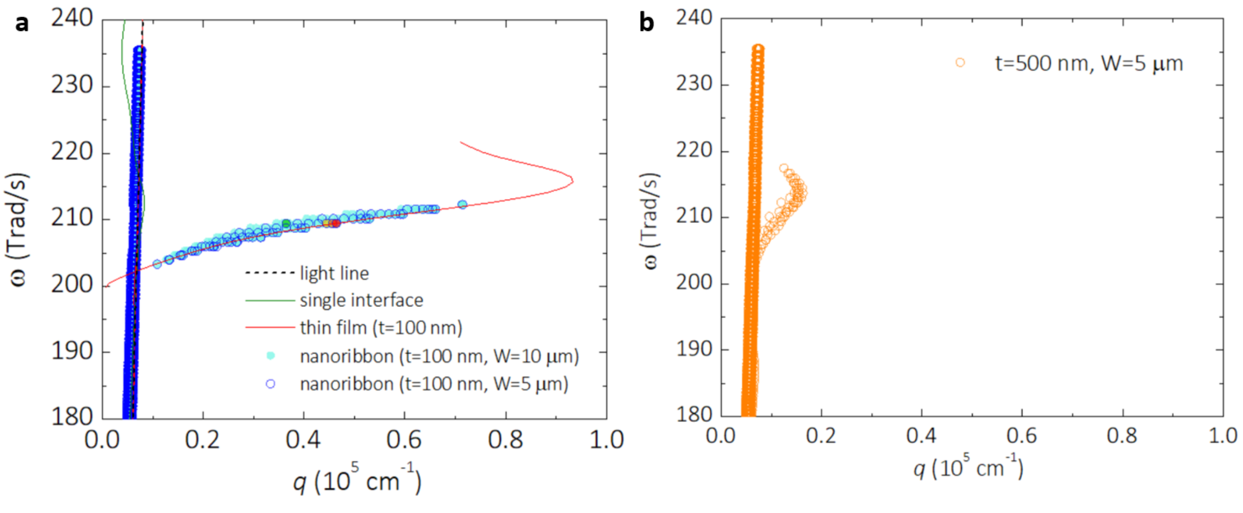

Supplementary Figure 23. Dispersions of nanoribbons by numerical modelling with (a) 100 nm and (b) 500 nm thickness.

Supplementary References

1 Cahill, D. G. Thermal conductivity measurement from 30 to 750 K: the 3ω method. *Review of scientific instruments* **61**, 802-808 (1990).

2 Golyk, V. A., Kruger, M. & Kardar, M. Heat radiation from long cylindrical objects. *Phys Rev E Stat Nonlin Soft Matter Phys* **85**, 046603, doi:10.1103/PhysRevE.85.046603 (2012).

3 Wuttke, C. & Rauschenbeutel, A. Thermalization via Heat Radiation of an Individual Object Thinner than the Thermal Wavelength. *Phys Rev Lett* **111**, doi:ARTN 024301 10.1103/PhysRevLett.111.024301 (2013).

4 Ingvarsson, S., Klein, L., Au, Y. Y., Lacey, J. A. & Hamann, H. F. Enhanced thermal emission from individual antenna-like nanoheaters. *Opt Express* **15**, 11249-11254 (2007).

5 Palik, E. D. Handbook of Optical Constants of Solids (Academic, Orlando, 1985). *Google Scholar*, 286-297.

6 Dai, S. *et al.* Tunable phonon polaritons in atomically thin van der Waals crystals of boron nitride. *Science* **343**, 1125-1129 (2014).
